# Transfer of Mycotoxins from Lactation Feed to Colostrum of Sows

**DOI:** 10.3390/ani10122253

**Published:** 2020-11-30

**Authors:** Paolo Trevisi, Diana Luise, Elisa Spinelli, Federico Correa, Elena De Leo, Giovanna Trambajolo, Giuseppe Diegoli, Paolo Bosi

**Affiliations:** 1 Department of Agricultural and Food Sciences (DISTAL), Alma Mater Studiorum-University of Bologna, Viale G. Fanin 46, 40127 Bologna, Italy; paolo.trevisi@unibo.it (P.T.); diana.luise2@unibo.it (D.L.); elisa.spinelli4@unibo.it (E.S.); federico.correa2@unibo.it (F.C.); 2 LAEMMEGROUP s.r.l., Via Vittime del Vajont 18, 10024 Moncalieri, Torino, Italy; edeleo@laemmegroup.it; 3 Collective Prevention and Public Health Service, Veterinary Health and Food Hygiene Area, Regione Emilia—Romagna, Viale A. Moro 21, 40127 Bologna, Italy; giovanna.trambajolo@regione.emilia-romagna.it (G.T.); giuseppe.diegoli@regione.emilia-romagna.it (G.D.)

**Keywords:** feed, colostrum, mycotoxin, sow

## Abstract

**Simple Summary:**

Mycotoxin risk levels are known to vary depending on the type of mycotoxins, animal species, age and type of production. In lactating animals, mycotoxins can be transferred from feed consumed to colostrum and milk. This has been well documented for aflatoxicosis in cattle, but not in sows and not for the most diffused mycotoxins. Samples of in-house produced complete feed for lactating sows were obtained from Italian farms. Samples of colostrum were obtained at farrowing from each farm. The feed content of the mycotoxins was generally low. Of the 19 samples analyzed, 10, 12, 17 and 2 were positive for aflatoxins, fumonisins, deoxynivalenol (DON) and zearalenone, respectively; however, only two were above the risk limit. The colostrum samples were all negative for aflatoxins while a faint analytical signal was detected for fumonisins in five samples, notwithstanding very low values in the feed. Deoxynivalenol was frequently present in the colostrum; the highest value was seen in the farm presenting the highest value in the feed. However, this colostrum value was far from the presumed risk value for suckling piglets. More controls regarding feed on the farm are suggested while more studies are advisable regarding the risk of DON toxicosis in suckling pigs.

**Abstract:**

Studies regarding the transfer of mycotoxins from sow feed to colostrum are scarce. A sample of in-house produced lactation feed and one of colostrum were collected from two or three sows per farm (total 49) from 19 farms. The feed contents of aflatoxins (AFs), fumonisins (FUs), deoxynivalenol (DON) and zearalenone (ZEA) were assessed using ELISA and confirmed by liquid chromatography-mass spectrometry (LC-MS), The values were very low (10, 12, 17 and 2 positive samples for AFs, FUs, DON and ZEA, respectively), except for two samples (one AF, one DON). Based on feed values, colostrum samples from 13 farms were tested for at least one mycotoxin (Total 35). Aflatoxins were not found in any sample. A signal for FUs was observed in 5 of 11 colostra, despite low feed values; DON was frequently present in the colostrum (10/14). On the farm where the feed exceeded the DON suggested limits, a higher colostrum content was seen, 10.9 µg/kg, approximately 1/69 of the value showing toxicity in young pigs. The absence of reference values for neonate pigs, and the risk of higher and longer ingestion of DON by sows suggested considering routine checks of sow feed; more research on DON transfer and toxicity in piglets is needed.

## 1. Introduction

Mycotoxins are produced by the secondary metabolism of some fungal species as a result of the environmental stress to which the plant has been subjected, or they develop during the drying process and conservation of plant products. Mycotoxins have been widely detected in several foods and feeds worldwide [1]. The problem of mycotoxins in animal husbandry is currently very concrete. It results in substantial economic losses related to the worsening of the productive performance of livestock animals as a result of acute, sub-acute or chronic diseases [2]. Numerous studies have characterized the main mycotoxins and their metabolites, and have shown their negative effects on animal and human health [3]. The most relevant groups of mycotoxins found in animal feed are produced by three genera of fungi: *Aspergillus* (aflatoxins (AF) and ochratoxin A), *Fusarium* species (trichothecenes (deoxynivalenol-DON), fumonisins (FU), and zearalenone (ZEA)) and *Penicillium* (ochratoxin A) [3]. In pigs, mycotoxicosis after the dietary ingestion of AFs, trichothecenes, FUs and ZEA is diffuse and may exert a negative effect on several organs, including the digestive tract [4], the immune [5], reproductive [6] and respiratory systems [7], the kidney [8] and the liver [9]. For these reasons, the European Commission has fixed maximum limit values for the content of AF B1 in complete feedstuffs ranging from 5 to 20 μg/kg depending on the target species, inside the general Directive concerning undesirable substances in feeds [10]. Furthermore, the European Commission set maximum guidance values of 0.9 to 5 mg/kg for DON, of 5 to 50 mg/kg FU B1 + B2 and of 100 to 500 μg/kg for ZEA in complete or complementary feeding stuffs [11].

The absorption of free mycotoxins present in the diet is generally a fast process [12,13] resulting in a diffuse presence in the blood circulation. The transmission of mycotoxins and their metabolites between animals (mother to newborns) can occur by means of colostrum and milk, the first sources of nutrients for piglets. In pigs, evidence is scarce regarding the risk posed by the mammary gland secretions to transfer mycotoxins to the offspring, as has already been abundantly pointed out for ruminants. In fact, numerous studies have clearly demonstrated the transfer of mycotoxins from feed to cow’s milk, especially for aflatoxins and its metabolites [14,15], since this milk is consumed directly or is processed by humans. The European Commission set a 0.050 μg AF metabolite M1/kg milk limit intended to regulate human consumption or the manufacturing of milk-based products [16].

It has been verified that a transfer of aflatoxins to sow milk occurs as indicated by the recovery of AF B1 or its metabolites M1 and M2 [17,18]; however, it is much lower than that detected in cows. Nevertheless, a Serbian survey [19] has found several samples of sow milk with values two or three times higher than that of the European Union (EU) limit fixed for cow’s milk destined for human consumption [16]. As for fumonisins, in the only trial carried out on sows, minimal detectable amounts of FU B1 were found in the milk during a two-week intake of FUs [20]. As for trichothecenes, and particularly for DON, the authors did not find any references in the literature regarding this topic. Moreover, studies on DON transfer from gut to milk in other species are also scarce. In women, it has been calculated that a single dose greater than the tolerable daily intake (TDI), equal to one microgram per kg of live weight, resulted in DON transfer into the milk [21], representing a risk for the breastfed infant. The same study showed that, when doubling the dose of exposure of the mother, the impact on the newborn (diarrhea, vomiting, immunosuppression) may involve its overall development [21].

However, data which tested the association between sow feed and milk are scarce. The aim of this study was to investigate whether the intake of mycotoxins before parturition led to their transfer to the colostrum as a potential source of risk for newborns. For this purpose, it was preventively agreed to concentrate the study on farms where in-house feed mixture was used for sow feeding. In fact while in industrial plants for feed production, controls regarding the presence of mycotoxins in raw materials are rather routine, feedstuff controls at the farm level are usually scanty in Italy; moreover, in farm feed mixing plants, the environmental parameters (e.g., humidity) are poorly controlled. Therefore, for these reasons, it was assumed that the risk for contamination from mycotoxins could be great in in-farm final feed mixtures. At the same time, the study was an opportunity for verifying the level of contamination of the main mycotoxins in in-house produced feed mixtures for sows. 

## 2. Materials and Methods 

### 2.1. Sampling of Feed and Colostrum

The experimental protocol, involving sows reared under conventional farm conditions, complied with European Council Directive 2008/120/EC. Before the experiment began, the protocol was explained to the farm owners. No procedure under the current law was applied to collect the colostrum. 

Samples of the feed mixture for lactating sows were obtained from 19 farms located in the Emilia-Romagna, Lombardy and Veneto regions. The farms selected complied with all the following characteristics: 1) they produced an in-house feed mixture, as defined in the preliminary aim; 2) they administered the same batch of feed for at least 5 days before farrowing until the time of sampling (1st day of lactation); 3) they did not use chelating agents. The second condition was necessary in order to ensure that the feed had already been metabolized by the sows for some days and, therefore, there was no interference with a feed administered previously. The third condition was to select farms which did not use additives aimed at binding, degrading or reducing the absorption of mycotoxins in the diet. 

Regarding the formula of the feed mixture, it was not possible obtain the same mixture on all farms. However, the formulas were generally based on the most common raw materials for sow lactation feed (barley, corn, soybean meal, wheat by-products) and were designed to comply with standard feeding requirements [22]. The feed mixture in the last week pre-farrowing was, in general, supplied following the typical standards for this phase, approximately 3.2 kg per day; however, this amount was mildly restricted on the day before the predicted farrowing. No systematic control of the feed residuals was carried out on the day of farrowing. All the feed mixtures were in milled form and not in pellets.

One day post-farrowing, colostrum was obtained from 2 or 3 multiparous sows from each farm for a total of 49 individual colostrum samples. At least 10 ml of colostrum were sampled by manual milking of each sow from at least four mammary glands directly into test tubes which were immediately frozen and preserved at −20 °C. The sows were not treated with oxytocin before the sampling, and it was not necessary to separate newborn pigs from the sows since, within the first day post farrowing, colostrum is also easily obtained in the presence of offspring.

The mycotoxin contents (AF B1/B2/G1/G2, FUs, DON and ZEA) were assessed on all 19 samples of feed collected. Based on the contents of each mycotoxin found in the feed of each farm, the colostrum collected in 13 out of 19 farms were then selected for the analyses, for a total of 35 samples. Specifically, 13, 12 and 14 of these samples were analyzed for AFs, FUs and DON, respectively. No colostrum sample was analyzed for ZEA due to the absence of feeds positive for this mycotoxin.

### 2.2. Methods of Analysis

The content of AF B1/B2/G1/G2, FUs, DON and ZEA in the feeds was assessed using commercial enzme-linked immunosorbent assay (ELISA) kits on a Multiskan multiplate reader (Multiskan™ FC Microplate Photometer—Thermo Fisher Scientific, Rodano (MI), Italy). The highest and a few low mycotoxin values for a total of 11 results were then validated using liquid chromatography-mass spectrometry (LC-MS). The analyses were carried out at LAEMMEGROUP lab (Moncalieri, Torino, Italy) with certified procedures (Accredia, accreditation n. 0198). The values for the colostrum samples were obtained using LC-MS. Briefly, the essential aspects of the used methods are reported.

#### 2.2.1. Determination of Mycotoxins Using an ELISA Kit

Aflatoxins—A competitive enzyme immunoassay for the quantitative determination of total aflatoxins using a BIO-SHIELD TOTAL kit (Prognosis Biotech, Larissa, Greece) (limit of quantification for livestock products: 1 μg/kg) was carried out. The toxins were extracted from 5 g of sample with 25 mL of a 70% methanol solution. The standard solutions and samples were added to the wells. The measurement of the intensity of the yellow color was carried out photometrically at 450 nm, and this intensity was inversely proportional to the concentration of total aflatoxins present in the sample. 

Fumonisins—A competitive enzyme immunoassay for the quantitative determination of FUs using a BIO-SHIELD FUMONISIN kit (Prognosis Biotech) (limit of quantification: 150 μg / kg) was carried out. Toxins present in 5 g of sample were extracted with 25 mL of a 70% methanol solution. With the substrate Chromogen in all wells, a progressive blue color developed and the addition of an acid solution allowed the color to change from yellow to blue. The measurement of the intensity of the yellow coloring was carried out photometrically at 450 nm and this intensity was inversely proportional to the fumonisin concentration present in the samples. 

Deoxynivalenol—A competitive enzyme immunoassay for the quantitative determination of DON using an ELISA kit for the determination of DON Celer DON v3 (Tecna, Trieste, Italy) (limit of quantification 125 μg / kg) was carried out. The toxin was extracted from a 50 g sample with 250 mL of a 70% methanol solution. After blocking the enzymatic reaction, the absorbance was measured using a colorimetric reader of microplates equipped with a 450 nm filter; the color development was inversely proportional to the concentration of DON in the sample. 

Zearalenone—A competitive immunoassay for the quantitative determination of ZEA using a BIOSHIELD ZON kit (code B2748 or B2796, produced by Prognosis Biotech) (Limit of quantification: 25 μg/kg) was carried out. For the extraction of the toxin, 50 g of sample were weighed, and 250 mL of a 70% methanol solution were added; the extract was filtered and collected in a container. The filtered extract was used for the execution of the kit. With the substrate Chromogen in all wells, a progressive blue color was developed, and the addition of an acid solution allowed the color to change from blue to yellow. The measurement of the intensity of the yellow color was carried out photometrically at 450 nm.

#### 2.2.2. Determination of Mycotoxins Using LC-MS 

Aflatoxins—Five g of sample were extracted with 20 mL of methanol:water (60:40, v/v); the extract was diluted with phosphate buffer solution (PBS) and purified using an immunoaffinity column. The eluate, dried and taken up with a mobile phase, was analyzed using UHPLC on Hypersil GOLD column C18, combined with Thermofisher TSQ 80000 (Thermo Fisher Service, Parma, Italy) quantitative mass spectrometer in selective reaction monitoring (SRM) mode. The aflatoxins B1, B2, G1 and G2 were identified using retention time verification and the recognition of characteristic m/z ratios. 

Fumonisins—The FUs were extracted from the sample with an additional mixture of solvent passages (HCl mixture 0.1 M/Acetonitrile 80:20 (v/v); HCl 0.1 M; methanol). The extract was diluted with PBS and purified using an immunoaffinity column. The eluate, dried and taken up with a mobile phase, was analyzed using UHPLC on a Hypersil GOLD C18 column and mass spectrometer in SRM mode. The fumonisins B1 and B2 were identified by checking the retention time and recognizing the characteristic m / z ratios. 

Deoxynivalenol—The extracted DON was analyzed using UHPLC on Kinetex Biphenyl and mass spectrometer in SRM mode with a negative ionization mode. The eluents were 0.1% formic acid, 0.1% formic acid in acetonitrile and wash―water mixture/methanol (50:50) (v/v). The DON was identified by checking the retention time and the recognition of the characteristic m/z ratios.

### 2.3. Statistical Methods

The results are presented as individual values for mycotoxin content in the feed of each farm and of individual colostrum samples.

Regarding the total number of feed mixtures for the sows, the frequencies of the samples with mycotoxin values above the quantification threshold (0.0010 mg/kg; 0.125 mg/kg; 0.15 mg/kg; 0.025 mg/kg for AFs, DON, FUs and ZEA, respectively) and above the maximum level allowed in the EU for AF [10] or the maximum guidance values recommended by the EU for the other three mycotoxins [11] were calculated.

For the DON and FU simple correlation coefficients between the in farm values and the values in the colostrum were also calculated (PROC REG of SAS, SAS Institute Inc., Cary, NC, USA), considering the values found in the colostrum under the detection limit to be equal to zero. For the FUs in the feed, the sum of the B1 and the B2 values was used, when available. 

## 3. Results and Discussion

**Figure 1:**
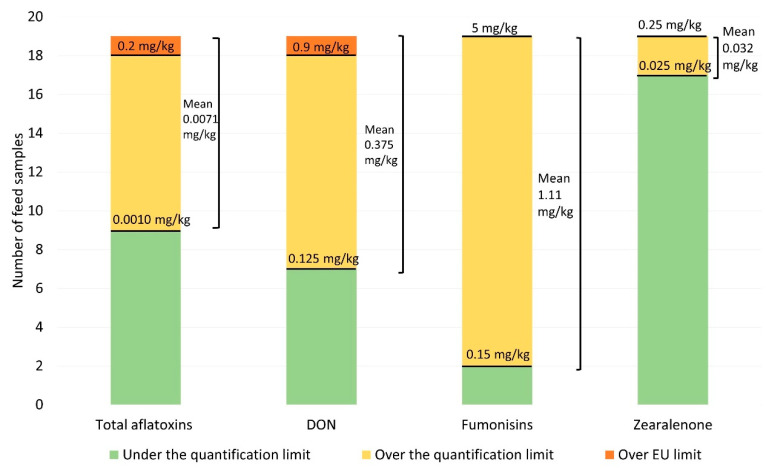
Frequencies of the samples of feed mixtures for sows, showing mycotoxin values above the quantification threshold (indicated as lower value per each histogram) and above the limits set by EU [10,11] (upper values), and means of values for each mycotoxin.

Table 1 presents the mycotoxin contents in the feed mixtures which were sampled for the lactating sows. Of the 19 samples collected, the levels of the mycotoxins detected were globally low (numbers of positive samples: 10, 12, 17 and 2 for AFs, FUs, DON and ZEA, respectively), and were always within the limits of the EU directives or the EU recommendations, except for two samples (one for AFs, one for DON) (Figure 1). Nevertheless, the presence of two farms, each having one mycotoxin value over the limit pointed out the need for controls on in-farm produced sow feeds.

All the colostrum samples collected from the 13 farms selected were tested for at least one category of mycotoxin (AFs; FUs; DON) and results are presented in Table 2. All the colostrum samples were negative for AFs. The concentration of AFs found in the feed samples was apparently too low to detect their presence in the colostrum, considering that, in other studies carried out on sows a transfer to milk was highlighted at higher dosages in the feed. In fact, the level of aflatoxins, AFB1 and AFB2 were 6.4 ± 0.5 and 0.67 ± 0.05 μg/kg of feed, respectively [16]. These data showed that the transfer levels (ratio between AF values in colostrum or milk, and AF values in feed) were very low, while, for AFB1 and AFM1, they were less than 0.1%, and for AFB2, it was 0.35% [10]. Feeding sows a gestating–lactating diet based on corn contaminated with AFB1 and FU B1 (130 and 5200 μg/kg diet, respectively) from the 80th day of gestation, resulted in a transfer of AFs of 0.33% for colostrum and 0.20% for milk [23]. If the percentage of excretion in sow milk is compared with that observed for the cow, which ranges from 0.5% to 5% [14,15], those values are much lower.

Nevertheless, high values of AFM1 can be found where controls of raw materials are less stringent. In a survey, carried out using the ELISA method, on ten farms selected because they were critical for AF occurrence in the feed or for a sow health condition, Prodanov-Radulović et al. [19] observed that 97% of the milk samples collected at 3–5 days of lactation were above the limit of determination for AFM1. Furthermore, half of the farms tested presented AFM1 values above 60 ng/L.

In this trial, the selection criterion for FUs was the same as that adopted for AFs. Despite being present in small quantities in the feed, a signal in the colostrum was often detected (5/11 of colostrum samples); however, in general, under the standard quantification limit (LOQ). As a rule, positive colostrum is associated with the feed having the highest concentration of FUs. This indicates that, for this mycotoxin, attention should be paid to the feed ingredients for sows. However, the authors did not find a statistically significant correlation between the presence of DON in the feed given in the immediate pre-farrowing period and that seen in the colostrum of sows (*r* = +0.301 *p* = 0.341, data not in a table). The reduced transfer to the colostrum is justifiable considering the reference values given in another study carried out on the effects of fumonisins [20]. The transfer of fumonisins into the milk was observed only after long periods of intake of feed contaminated with this mycotoxin. Diets containing 2–3 mg of FUs per kg of feed did not show a consistent transfer to milk and muscle. In fact, sows fed for 14 days with a diet containing 100 mg of FUs per kg of feed showed a transfer rate to milk which was below the 30 parts per billion (ppb) threshold [20]. The observations regarding sows were also indirectly confirmed by the carry-over values seen in milk from cows [24,25,26]. Overall, the possibility of a high rate of ingestion of FUs with colostrum by piglets seemed rather modest. However, no data are available on the effect regarding the health of suckling pigs chronically exposed to a low dose of FUs. 

The data produced in this study demonstrated the diffusion of DON in the feedstuffs used for lactating sows, with values closer to the limit suggested for the feed. The ability of DON to be transferred from the bloodstream to the colostrum was also demonstrated, shown in 10 out of 14 of the colostrum samples tested. The coefficient of correlation between the values in the feed and those in the colostrum was +0.647 (*p* = 0.012). This overall value indicated that there was an evident association between the presence of DON in the feed given in the immediate pre-farrowing period and that seen in the colostrum of the sow which, to the best of the authors’ knowledge, had not previously been evidenced.

In particular, the farm presenting the feed sample contaminated with the DON value higher than the suggested limit presented the highest level of DON in the colostrum (10.9 µg/kg). In this regard, as far as the authors know, there are no indications regarding the transfer of DON to sow milk and on the possibility that the toxic limits for piglets were reached when contaminated colostrum or milk was suckled. However, an estimation can be carried out considering the highest value found in the sow colostrum in the present study and in toxicity studies for DON in weaned piglets of 4 weeks of age. In particular, it was demonstrated that a chronic intake of doses ranging from 2.5 to 3.5 mg/kg feed over a 5 to 9 week period severely affected the intestinal homeostasis by decreased villus height and reduced mucosa integrity, and upregulated several genes related to inflammation [27,28]. Upon antigenic stimulation (Ovalbumin), the antibody response, particularly for immunoglobulins A, the lymphocyte proliferation and the cytokine expression in the lymph nodes were altered [27,28,29]. Therefore, considering 2.5 mg/kg feed of DON as the minimum value for the presence of mycotoxicosis in the piglet diet, and considering the ratio of 1/3 between the dry matter of porcine colostrum (27%) [30] and reference feed (88%), the concentration of DON in the colostrum at reference dry matter was 0.036 mg/kg, i.e., 1/69 of the value showing toxicity in young pigs. The DON value corrected for reference dry matter was also 1/25 of the maximum guidance values set for all pigs by the European Union [11]. In the only study in which litter performance was controlled [31], when gilts received up to 6.2 mg/kg (from contaminated wheat) in the gestation-lactation diets, no adverse effect was seen on the litter growth as compared to a control diet. Interestingly, the short-term intrauterine exposure of sows to pure DON intravenously administered during gestation induced a long-term persistence of DON in the plasma of the offspring, and caused an alteration in the immune parameters [32]. Nevertheless, the absence of reference values for the toxicity level in newborn pigs, their reduced immunocompetence [33], the risk of the higher ingestion of DON due to continuous milk intake and the potential ingestion of contaminated feed during the entire lactation period by sows, suggested controlling the sow feed frequently, and more research, particularly regarding DON transfer and toxicity in the piglet, is needed.

## 4. Conclusions

The values found for the presence of aflatoxins in feed mixtures given to sows in the immediate days pre-farrowing did not pose a risk of transfer to the colostrum since aflatoxins were not detectable in the colostrum samples. Feed samples showing the presence of fumonisins greater than the maximum guidance values were rare. However, more attention should be paid to fumonisin levels regarding transfer to the colostrum as a potential source of risk for newborns. For DON, the absence of reference values for suckling pigs suggested controlling the sow feed frequently and carrying out more research, particularly for DON transfer from the mother to the colostrum or milk, and for toxicity in the suckling piglet. Zearalenone contamination of in-house produced complete sow feed used in the selected Italian farms did not seem to pose problems.

## Figures and Tables

**Table 1:** Mycotoxin content in the feed mixtures which were sampled for sows (mg/Kg at 88% DM, using ELISA or validated using LC-MS when marked by *).

Farm	Total Aflatoxins	DON	Fumonisins	Zearalenone
1	<0.0010	0.438	1.52	<0.025
2	<0.0010	0.110	0.37	<0.025
3	0.0070	0.101	1.02	<0.025
4	0.0014	0.164	0.24	<0.025
5	0.0190	<0.125	2.59	<0.025
6	<0.0010	<0.125	<0.15	<0.025
7	0.0220	0.184	3.74	0.043
8	0.0015	0.214	0.56	<0.025
9	<0.0010	<0.125	2.9	<0.025
10	<0.0010	0.218	0.29	<0.025
11	0.0033	<0.125	2.36	<0.025
12	<0.0010	0.60 *	0.26	<0.025
13	0.0091	<0.125	1.3	<0.025
14	<0.0010 *	1.17 *	0.06 FB1 * 0.05 FB2 *	0.02 *
15	0.0022	<0.125	0.58	<0.025
16	<0.0010	0.67 *	<0.15	<0.025
17	0.0006 ^1^ *	0.133 *	2.96 FB1 * 0.58 FB2 *	<0.025 *
18	0.0022	0.172	0.22 FB1 * 0.10 FB2 *	<0.025
19	0.0033 *	<0.01 *	0.85	<0.025

^1^ Present signal for B1 lower than the limit of quantification (LOQ).

**Table 2:** The concentrations of mycotoxins observed in sow colostrum sampled from the different farms using LC-MS; the samples were chosen in order to represent the range of values found in the respective in-home feed mixtures. Values represent each colostrum sample from an individual farm. LOQ = limit of quantification. All the samples from farms 3, 7, 11, 17 and 19 were negative for aflatoxins.

Farm	DON (µg/kg)	Farm	Fumonisins (µg/kg)
8	<10.0	9	8.6 (signal for FB1 < LOQ) no FB2
8	1.7 (signal < LOQ)	9	<20,0 FB1 and FB2
8	1.2 (signal < LOQ)	9	5.7 (signal for FB1 < LOQ) no FB2
10	1.1 (signal < LOQ)	14	<20.0 FB1 and FB2
10	<10.0	14	<20.0 FB1 and FB2
10	<10.0	15	<20.0 FB1 and FB2
10	1.0 (signal < LOQ)	15	<20.0 FB1 and FB2
12	2.0 (signal < LOQ)	15	<20.0 FB1 and FB2
12	1.3 (signal < LOQ)	17	<20.0 FB1 and FB2
12	1.2 (signal < LOQ)	17	1.3 (signal for FB1 < LOQ) no FB2
14	1.7 (signal < LOQ)	18	3.2 (signal for FB1 < LOQ) no FB2
14	10.9	18	3.7 (signal for FB1 < LOQ) no FB2
16	4.0 (signal < LOQ)	-	-
16	<10.0	-	-
